# Atypical alpha oscillatory EEG dynamics in children with Angelman syndrome

**DOI:** 10.1016/j.nicl.2025.103865

**Published:** 2025-08-13

**Authors:** Abigail H. Dickinson, M.Sapphire Bowen-Kauth, Jeremy J. Shide, Anna E. Youngkin, Nishitha S. Hosamane, Courtney A. McNair, Declan P. Ryan, Catherine J. Chu, Michael S. Sidorov

**Affiliations:** a Psychiatry and Biobehavioral Sciences, University of California, Los Angeles, USA; b Center for Neuroscience Research, Children’s National Hospital, Washington, DC, USA; c Department of Neurology, Massachusetts General Hospital, Boston, MA, USA; d Harvard Medical School, Boston, MA, USA; e Departments of Pediatrics and Pharmacology & Physiology, The George Washington University School of Medicine and Health Sciences, Washington, DC, USA

**Keywords:** Angelman syndrome, EEG, Alpha, Biomarker, Oscillatory dynamics

## Abstract

**Objectives:**

Biomarkers of atypical brain development are crucial for advancing clinical trials and guiding therapeutic interventions in Angelman syndrome (AS). Electroencephalography (EEG) captures well-characterized developmental changes in peak alpha frequency (PAF) that reflect underlying neural circuit maturation and may provide a sensitive metric for mapping atypical neural trajectories in AS.

**Method:**

We analyzed 159 EEG recordings from 95 children with AS (ages 1–15 years) and 185 age-matched typically developing (TD) controls. PAF was quantified using a well-established curve-fitting method applied to 1/f-corrected power spectra. To validate robustness, we further evaluated PAF using an alternative prominence-based peak detection approach across varying detection thresholds.

**Results:**

Significant disruptions in PAF were evident in children with AS. While over 90% of EEGs from TD children exhibited a clear alpha peak, fewer than 50% of EEGs from children with AS showed a detectable PAF. Furthermore, when PAF was present, its frequency was significantly lower in AS children and did not show the typical age-related increases observed in TD children. Validation analyses confirmed consistently lower rates of PAF detection in AS across varying sensitivity thresholds, demonstrating the robustness of these results.

**Conclusions:**

The absence and lower frequency of alpha peaks in Angelman syndrome indicate that PAF is a developmentally sensitive marker of disrupted neural maturation in this population. Further research is needed to clarify how PAF emergence and shifts relate to longitudinal developmental trajectories and specific clinical phenotypes. Nonetheless, PAF shows promise as an objective, quantitative biomarker of neural circuit dynamics that can enhance clinical‐trial endpoints by indexing underlying brain function. Future analyses will examine inter‐individual variability in PAF among AS participants to uncover mechanistic pathways that may inform targeted therapeutic strategies.

## Highlights

• EEG-derived PAF is a marker of neural maturation in childhood.
• PAF is significantly disrupted in children with Angelman syndrome.
• Fewer AS children showed a clear PAF compared to TD peers.
• When present, PAF was significantly lower in AS and did not increase with age.
• PAF may improve outcome measures in Angelman syndrome clinical trials.

## 1 Introduction

Angelman syndrome (AS) is a rare neurodevelopmental disorder characterized by significant cognitive and motor impairments, limited or absent speech, seizures, sleep disruptions, and pervasive developmental delays (Bird, 2014, Williams, 2005). These clinical features result from the loss of function of the maternal copy of the ubiquitin-protein ligase E3A (*UBE3A*) gene, typically due to deletions or mutations (Kishino et al., 1997). In neurons, the paternal *UBE3A* allele is silenced by the long, non-coding antisense transcript *UBE3A-ATS*, making the maternal allele the sole source of UBE3A protein (Meng et al., 2012, Rougeulle et al., 1998, Runte et al., 2001). The resulting UBE3A deficiency in AS disrupts neuronal and synaptic processes essential for early brain development, leading to the defining clinical characteristics of the disorder.

Preclinical research has leveraged the conserved paternal imprinting of UBE3A in mice (Albrecht et al., 1997, Judson et al., 2014) to explore treatments aimed at reactivating the paternal allele. These efforts have shown remarkable efficacy in AS mouse models (*Ube3a^m-/p+^*) (Huang et al., 2011, Meng et al., 2013, Meng et al., 2015, Milazzo et al., 2021, Vihma et al., 2024, Wolter et al., 2020), and have advanced to clinical trials (Elgersma and Sonzogni, 2021). Alongside preclinical progress in other mechanism-based therapies (Copping et al., 2021), these developments underscore the urgent need for robust biomarkers to evaluate and guide therapeutic interventions in AS (Berry-Kravis et al., 2018).

Electroencephalography (EEG) is a non-invasive, accessible, and scalable method for measuring brain activity that holds significant potential for identifying such markers. EEG provides a direct measure of brain activity and can capture subtle oscillatory dynamics targeted by disease-modifying therapies, offering a sensitive means to evaluate target engagement and therapeutic effects in real-time. EEG has been widely used to investigate markers of atypical neural function in neurodevelopmental disorders, including AS (Goodspeed et al., 2023). One of the most well-documented EEG findings in AS is an increase in low-frequency (∼1–4 Hz) delta rhythms (Bower and Jeavons, 1967, Boyd et al., 1988, Laan et al., 1997, Vendrame et al., 2012), an observation that has recently been supported by quantitative studies (Frohlich et al., 2019, Levin et al., 2022, Martinez et al., 2020, Sidorov et al., 2017). Increased delta in AS correlates with clinical severity (Hipp et al., 2021, Ostrowski et al., 2021), and is now being evaluated as a biomarker in clinical trials. However, while delta power is a crucial marker of generalized neural disruption, it may lack sensitivity to detect more nuanced changes in cortical circuit development or therapeutic gains.

Alpha oscillations (6–12 Hz) may serve as a complementary biomarker for mapping circuit-level changes in AS. These rhythms play a crucial role in neuronal timing, functional inhibition, and integrating information across brain regions (Foxe and Snyder, 2011, Jensen and Mazaheri, 2010, Klimesch et al., 2007) and exhibit well-established developmental changes that are closely linked to cortical circuit maturation and cognitive function (Freschl et al., 2022, Marshall et al., 2002). During typical development, a distinct peak in alpha frequencies (peak alpha frequency; PAF), emerges towards the end of the first year (∼6 Hz) and gradually increases in frequency throughout childhood, reflecting the maturation and growing efficiency of neural circuits (Freschl et al., 2002).

The developmental trajectory of alpha oscillations provides a valuable benchmark for identifying and tracking atypical brain development. For instance, deviations in PAF emergence and maturation have been observed in autism, Fragile X syndrome, and neurofibromatosis type 1 (Booth et al., 2023, Dickinson et al., 2018, Pedapati et al., 2022). Furthermore, developmental changes in PAF are correlated with non-verbal cognitive abilities (Carter Leno, V., Pickles, A., van Noordt, S., Huberty, S., Desjardins, J., Webb, S.J., Elsabbagh, M., Team, B et al., 2021, Dickinson et al., 2019, Edgar et al., 2019), suggesting that PAF may serve as a direct index of the circuit-level changes underlying cognitive function—a core therapeutic target in AS clinical trials. Despite this potential, alpha oscillations in AS have not been rigorously analyzed across a broad developmental window. While prior studies have presented power spectra suggesting likely alpha alterations in AS (Frohlich et al., 2019, Ostrowski et al., 2021, Sidorov et al., 2017), it remains unclear whether alpha metrics, such as PAF, sensitively index disrupted network dynamics or exhibit an atypical age-related trajectory in this population.

This study addresses this gap by characterizing alpha dynamics, specifically power and PAF, in children with AS and comparing these metrics to an age-matched typically developing (TD) cohort. Additionally, we examine age-related changes in alpha dynamics across the two groups. By providing a detailed assessment of these metrics, we aim to evaluate their potential as biomarkers of brain development in AS. Given the extensive neural disruptions associated with AS, we hypothesize atypical alpha patterns, including reduced power and frequency, as well as deviations in age-related trajectories.

## 2 Methods

### 2.1 Participants

This study utilized deidentified EEGs (n = 159) from children with AS and typically developing (TD) controls (n = 185), drawn from established repositories and previously published datasets (Table 1). Participants ranged in age from 0.9 to 14.9 years (TD: 5.8 ± 0.3 years; AS: 6.1 ± 0.3 years; Fig. S1a).

**Table 1:** **Participant demographics.** TD: typically developing, AS: Angelman syndrome.

*Group*	*# of EEGs*	*Unique participants*	*Age (years)*	*Male*	*Female*	*Length of EEG (minutes)*
TD	185	185	5.8 ± 0.3	99 (54 %)	86 (46 %)	10.3 ± 1.3
AS	159	95	6.1 ± 0.3	102 (64 %)	57 (36 %)	24.7 ± 2.1

A total of 159 EEGs from individuals with AS were accessed through established repositories. Of these, 149 EEGs were collected as part of the AS Natural History Study (ClinicalTrials.gov identifier: NCT00296764) and shared via the LADDER database (Potter et al., 2024), and 10 were collected by the UNC Sleep Disorders Center (Levin et al., 2022). These recordings represented 95 unique participants, as 35 individuals contributed multiple recordings (Supplementary Tables 1-2). Genetic information was available for 89 of the 95 AS participants, with a genotype distribution representative of the general AS population (Bird, 2014): 21 % class I deletion, 38 % class II deletion, 3 % atypical deletion, 6 % deletion (unspecified), 10 % paternal uniparental disomy, 4 % imprinting defect, 15 % UBE3A mutation, 3 % abnormal DNA methylation (Fig. S1b). Data on medication use at the time of EEG were available for 149/159 AS EEG recordings, representing 88 unique participants. Among those with available information, most participants (76/88) were reported to be taking at least one medication. A full list of reported medications is provided in the Supplementary Information.

Age-matched EEGs from TD controls were drawn from previously published datasets collected at three sites: Massachusetts General Hospital (MGH) (*n* = 98), UNC Sleep Disorders Center (*n* = 10), and UCLA (*n* = 77) (den Bakker et al., 2018, Dickinson et al., 2018, Dickinson et al., 2025, Levin et al., 2022, Ostrowski et al., 2021, Sidorov et al., 2017) (Supplementary Table 3). Children in these datasets were identified as TD by the studies through which their data were collected, based on the absence of neurodevelopmental or genetic conditions.

### 2.2 EEG processing

EEG data in the present analyses were derived from continuous (task-free) recordings pooled from multiple studies and sites. The specific system hardware configurations and recording parameters are detailed in Supplementary Tables 2 & 3**.** To address these variations, we applied data processing procedures designed to harmonize datasets and ensure consistent analysis across studies (Levin et al., 2022, Sidorov et al., 2017).

For clinical EEGs, periods of wake and sleep were identified and annotated by a trained neurologist, ensuring that only wakeful data were analyzed. Raw EEGs were preprocessed using methods adapted from prior studies (Levin et al., 2022, Sidorov et al., 2017) and implemented using custom MATLAB scripts. All recordings were processed using identical procedures. After raw EEG data were imported into MATLAB, data were re-referenced to the common average of all channels recorded (see Supplementary Tables 2 & 3 for a list of recorded channels by site). We then applied a 1 Hz high-pass and 99.5 Hz low-pass filter using a second-order Butterworth design, and a 60 Hz notch filter (stopband: 59–61 Hz).

Recordings were visually inspected to identify and exclude artifactual sections of data and channels with excessive noise. After artifact removal, an average of 17.0 ± 1.2 min of EEG data per recording underwent further analysis, with 10.3 ± 1.3 min for TD EEGs and 24.7 ± 2.1 min for AS EEGs (Table 1). All recordings retained at least 60 s of artifact-free data. To account for variability in the amount of data collected across participants, recording length (in minutes) was included as a covariate in all statistical models. This ensured that differences in peak detection or frequency estimates were not attributable to differences in recording duration.

To ensure consistency, analyses focused on six specific channels representing three regions of interest: F3 and F4 (frontal), C3 and C4 (central), and O1 and O2 (occipital). Across all recordings (2064 total channels), 28 channels were excluded (1.35 %), including 21 from AS recordings and seven from TD recordings. Missing channels affected 6.1 % of participants (17 AS, 4 TD). If both channels for a region were missing, the region was excluded from analyses for that participant. This impacted 1.45 % of participants (4 AS, 1 TD). Among these, four AS participants had one region excluded (3 frontal, 1 occipital), and one TD participant had two regions excluded (frontal and occipital).

### 2.3 Spectral power analysis

Spectral power was calculated separately for each channel and averaged across paired channels within each region of interest: F3/F4 (frontal), C3/C4 (central), and O1/O2 (occipital). Power spectra were computed using Welch’s method, applied to 2-second epochs with 50 % overlap, resulting in a frequency resolution of 0.5 Hz. The final power spectra represent the median across all epochs. Absolute power values were normalized to relative power by dividing the power at each frequency by the total power across the analyzed range (1–50 Hz). Band-specific values were then computed by summing relative power values within the delta (2–4 Hz) and alpha (6–12 Hz) frequency bands. The alpha band was defined as 6–12 Hz to accommodate the lower-frequency peaks characteristic of developmental and neurodevelopmental populations (Dickinson et al., 2025). Absolute power values are presented in Fig. S2; however, all analyses in this study were conducted using relative power. Relative power was analyzed, following standard conventions in developmental EEG research, particularly given the use of both clinical and high-density research systems with differing impedance properties (Supplementary Table 3).

#### 2.3.1 Peak alpha frequency

Peak alpha frequency (PAF) was quantified separately for each of the six channels of interest using a custom MATLAB script designed to isolate periodic alpha activity (Fig. S2a) (Dickinson et al., 2018). To remove bias toward lower spectral frequencies, the aperiodic 1/f signal was first subtracted (Neto et al., 2015). To identify the peak within the alpha range (6–12 Hz), a Gaussian curve was fitted to the spectra using the MATLAB *fit* function (Fig. S2b). If the curve-fitting procedure failed, this was taken as evidence of no significant modulation in the alpha frequency range. These cases were independently reviewed by two authors (MBK, AHD) to confirm the absence of a peak. If power modulation was present but could not be accurately fitted due to irregular spectral curves, the peak was identified manually. After quantifying peaks at the channel level, we calculated regional values for each of the three regions by averaging the peak values across the two channels within the region. If a peak was detected in only one channel, that value was used as the regional value.

#### 2.3.2 Prominence threshold analysis

Given the low rates of peak detection in AS spectra, we explored an alternative peak detection method to determine whether these low rates were influenced by the detection approach itself. While Gaussian fitting is designed to identify distinct and well-defined spectral peaks, it may fail to capture subtler features. To address this limitation, we implemented MATLAB’s *findpeaks* function, which identifies peaks based on prominence thresholds. Prominence is defined as the vertical distance between the peak height and the average power of the nearest troughs on either side (see Fig. 4a). Peaks with greater prominence are more distinct from surrounding power. Prominence-based analysis was conducted on power values normalized to a 0–1 range to ensure consistency across participants, with 25 logarithmically spaced thresholds values ranging from 0.02 to 1. By adjusting the prominence threshold across this range of values, this method offered greater flexibility in identifying a wider range of spectral features, including subtle peaks that Gaussian fitting might miss.

### 2.4 Statistical analysis

Mixed-effects models were implemented to account for the hierarchical structure of the data, addressing non-independence due to some participants contributing multiple EEGs, and accounting for missing data as needed. Participant ID was included as a random effect in all models to account for repeated measures within participants.

#### 2.4.1 Relative spectral power

Mixed-effects models were used to examine group differences in relative spectral power, specifically delta (2–4 Hz) and alpha (6–12 Hz) power, across frontal, central, and occipital regions. Fixed effects included group (AS vs. NT), age, and a Group × Age interaction, with participant ID included as a random intercept.

#### 2.4.2 PAF

To investigate differences in peak detection rates, a generalized linear mixed-effects model (GLMM) with a logit link function was used, with fixed effects for group, region, recording length, and age, as well as their interactions. For participants with detectable peaks, a linear mixed-effects model was fitted to analyze differences in PAF. PAF values were modeled using mixed-effects models with group (AS vs. NT), age, and a Group × Age interaction as fixed effects, recording length as a covariate, and participant ID included as a random intercept. Results are presented as mean ± SEM. Significance thresholds were defined as p < 0.05, p < 0.01, p < 0.001, and p < 0.0001, with corresponding annotations (*, **, ***, ****). For analyses in which three separate models were used to examine regional effects independently (i.e., alpha power, delta power, and predictors of peak frequency), family-wise error was controlled using a Bonferroni-adjusted significance threshold of p < 0.017 (0.05/3)

#### 2.4.3 Prominence-based peak detection

We used a GLMM to examine the relationship between group (AS vs. NT), threshold, and their interaction on the likelihood of peak detection across varying thresholds (n = 25). The model included fixed effects for group, threshold, and their interaction. At the median threshold value (0.14), we compared the primary Gaussian-based peak detection method with the secondary prominence-based analysis. Agreement in peak detection rates at this threshold was assessed using Cohen’s kappa. For participants with a detected peak at the median threshold, intraclass correlation coefficients (ICCs) were calculated to evaluate the consistency of peak frequency values between the two methods

## 3 Results

### 3.1 Increased delta power and decreased alpha power in AS

**Fig. 1:**
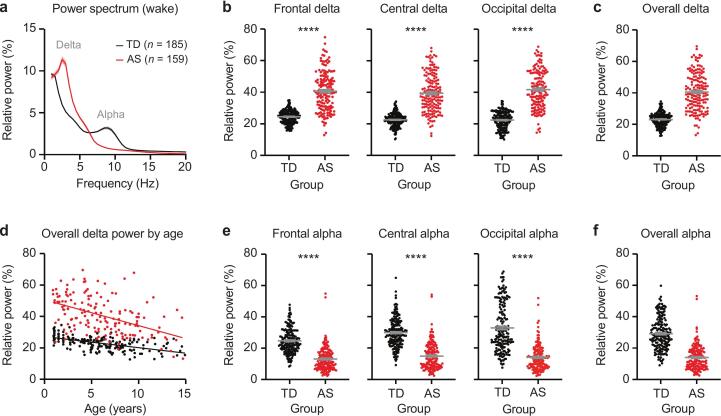
**Children with AS have increased delta power and decreased alpha power**. (a) Power spectrum of EEGs from typically developing (TD) children and children with AS during wake. Shaded area indicates ± SEM. (b) Relative delta power (2–4 Hz) is increased in AS in frontal, central, and occipital channels. Frontal: TD: *n* = 184, AS: *n* = 156; Central: TD: *n* = 185, AS: *n* = 159; Occipital: TD: *n* = 183, AS: *n* = 157. (c) Relative delta power, averaged across all electrodes. (d) Relative delta power as a function of age in TD and AS EEGs. (e) Relative alpha power (6–12 Hz) is decreased in AS in frontal, central, and occipital channels. (f) Relative alpha power, averaged across all electrodes. Data plotted as mean ± SEM; ****p < 0.0001.

Linear mixed-effects models revealed significantly higher delta power (2–4 Hz) in Angelman syndrome (AS) compared to typically developing (TD) participants across all regions (Frontal: coefficient = -0.22, 95 % CI [-0.26, −0.18], p < 0.0001; Central: coefficient = -0.246, 95 % CI [-0.283, −0.209], p < 0.0001; Occipital: coefficient = -0.288, 95 % CI [-0.324, −0.251], p < 0.0001; Fig. 1**a-b**). Relative delta power averaged across all electrodes is illustrated in Fig. 1**c**. Significant main effects of age indicated age-related declines in delta power for all regions (Frontal: coefficient = 0.00072, 95 % CI [-0.0003, −0.001], p < 0.01; Central: coefficient = 0.00095, 95 % CI [0.00052, 0.00139], p < 0.0001; Occipital: coefficient = 0.0011, 95 % CI [0.00065, 0.00152], p < 0.0001). Additionally, significant Group × Age interactions (Frontal: coefficient = -0.001, 95 % CI [-0.001, −0.0007], p < 0.0001; Central: coefficient = -0.0013, 95 % CI [-0.0016, −0.0010], p < 0.0001; Occipital: coefficient = -0.0017, 95 % CI [-0.0021, −0.0014], p < 0.0001) showed that these age-related declines were more pronounced in AS compared to TD participants, suggesting steeper reductions in delta power with age in AS (results averaged across all electrodes illustrated in Fig. 1**d**).

Linear mixed-effects models indicated significantly lower relative alpha power (6–12 Hz) in AS compared to TD participants across all three regions (Frontal: coefficient = 0.133, 95 % CI [0.103, 0.162], p < 0.0001; Central: coefficient = 0.184, 95 % CI [0.149, 0.218], p < 0.0001; Occipital: coefficient = 0.159, 95 % CI [0.111, 0.207], p < 0.0001; Fig. 1**e**). Relative alpha power averaged across all electrodes is illustrated in Fig. 1**f**. Significant main effects of age revealed that alpha power increased with age in both groups (Frontal: coefficient = 0.00083, 95 % CI [0.00059, 0.00107], p < 0.0001; Central: coefficient = 0.00104, 95 % CI [0.00078, 0.00130], p < 0.0001; Occipital: coefficient = 0.00117, 95 % CI [0.00088, 0.00145], p < 0.0001). Group × Age interactions indicated that age-related changes in alpha power were comparable across the two groups in frontal and central regions, with a trend-level effect in the occipital region (Frontal: coefficient = -0.0001, 95 % CI [-0.00044, 0.00024], p = 0.565; Central: coefficient = -0.00029, 95 % CI [-0.00068, 0.00010], p = 0.143; Occipital: coefficient = 0.00062, 95 % CI [0.00010, 0.00114], p = 0.020). Group × Age interactions indicated that age-related changes in alpha power were comparable across the two groups in frontal and central regions, with a trend-level effect in the occipital region. Spectral analyses of raw delta and alpha power are reported in Fig. S3.

### 3.2 Reduced PAF presence in AS

**Fig. 2:**
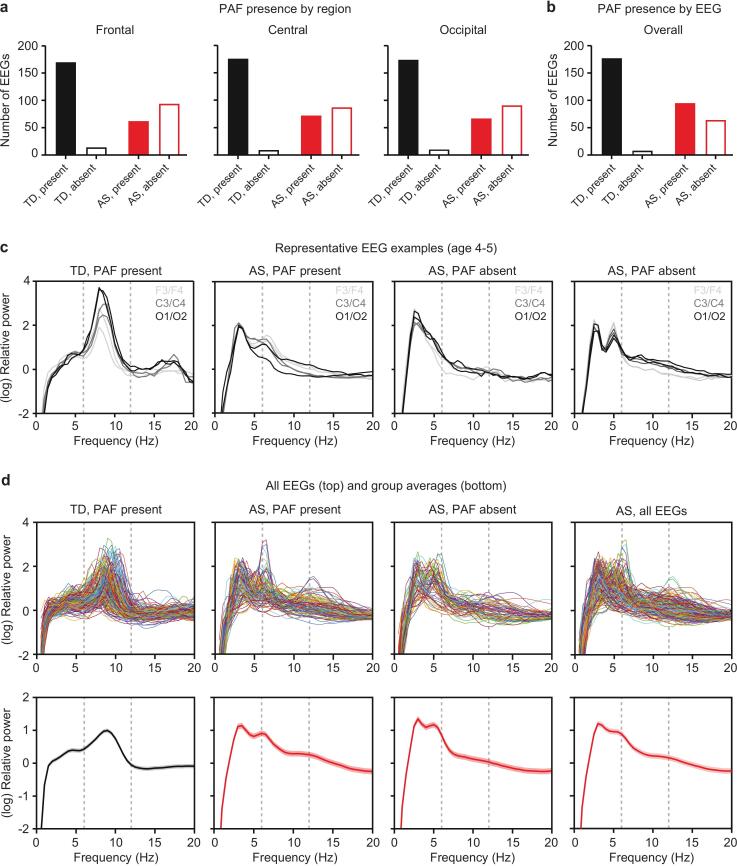
**PAF was not present in a subset of AS EEGs.** (a) PAF presence by region in TD and AS EEGs. (b) PAF presence by EEG in TD and AS EEGs. (c) Representative 1/f-removed spectra from four subjects illustrate typical detection of PAF in a TD EEG (left), qualitatively abnormal PAF in an AS EEG (second from left), absence of PAF in an AS EEG without (second from right) and with a theta peak outside the range of alpha (right). Six traces represent six channels in each plot. (d) Top panels show 1/f-removed spectra from all EEGs and bottom panels show group averages; shaded area indicates ± SEM. Dotted lines represent bounds of alpha (6–12 Hz).

In the AS group, 39.74 % of participants showed an alpha peak in the frontal region, 45.28 % in the central region, and 42.41 % in the occipital region. Comparatively, in the TD group, 92.39 % showed a peak in the frontal region, 95.14 % in the central region, and 94.57 % in the occipital region (Fig. 2**a**). When considering participants who showed a peak in any region, 59.75 % of the AS group displayed a peak, compared to 95.68 % in the TD group (Fig. 2**b**). To formally compare rates of alpha peak presence, we focused on regional-level data. A logistic mixed-effects model revealed a significant main effect of group (p < 0.0001), with TD participants being more likely to exhibit a peak compared to AS participants (log-odds = 3.59, 95 % CI [2.31, 4.87], Supplementary Table 4). A significant main effect of age (p < 0.0001) indicated that peak presence increased with age (log-odds = 0.018, 95 % CI [0.010, 0.026]). The non-significant Group × Age interaction (p = 0.91) suggests that this age-related increase was consistent across both groups. No significant differences in peak presence were observed between regions (p > 0.05), and Group × Region interactions were also non-significant (p > 0.05). Fig. 2 also illustrates 1/f-removed spectra across channels for four representative EEGs (1 TD; 3 AS; Fig. 2**c**), and individual traces for each EEG (Fig. 2**d**). The likelihood of peak alpha detection was not significantly affected by medication or AS genotype (Supplementary Tables 5–6).

### 3.3 Atypical developmental PAF trajectory in AS

**Fig. 3:**
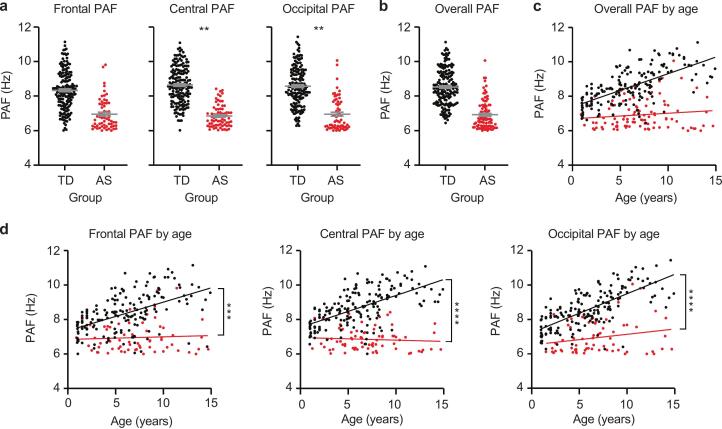
**PAF does not develop typically in AS EEGs.** (a) Comparison of PAF by group in frontal, central, and occipital regions. (b) Average PAF across all channels and (c) as a function of age. (d) PAF as a function of age in frontal channels, central channels, and occipital channels. Each point represents the averaged PAF of two channels per region of interest per subject. Error bars indicate ± SEM; **p < 0.01, ***p < 0.001, ****p < 0.0001. Asterisks in (d) represent group X Age interaction.

In EEGs where an alpha peak was present, linear mixed-effects models revealed a significant main effect of group in central and occipital but not frontal regions (Frontal: coefficient = 0.444, 95 % CI [-0.214, 1.102], p = 0.185; Central: coefficient = 0.746, 95 % CI [0.236, 1.256], p < 0.01; Occipital: coefficient = 0.738, 95 % CI [0.233, 1.244], p < 0.01), with TD participants showing higher PAF values compared to AS participants (Fig. 3**a,** Supplementary Table 7). PAF averaged across all electrodes is illustrated in Fig. 3**b-c**. There was a significant main effect of age indicating an age-related increase in PAF for the occipital but not frontal or central region (Frontal: coefficient = 0.0148, 95 % CI [-0.0563, 0.0859], p = 0.682; Central: coefficient = 0.00822, 95 % CI [-0.0473, 0.0637], p = 0.771; Occipital: coefficient = 0.0736, 95 % CI [0.0188, 0.128], p < 0.01). Significant Group × Age interactions in all regions indicated that TD participants exhibited significantly steeper age-related PAF increases (Frontal: coefficient = 0.152, 95 % CI [0.0696, 0.234], p < 0.001; Central: coefficient = 0.176, 95 % CI [0.111, 0.241], p < 0.0001; Occipital: coefficient = 0.152, 95 % CI [0.0872, 0.218], p < 0.0001; Fig. 3**d**). PAF was not significantly affected by medication or genotype (Supplementary Tables 8–9). For participants with multiple EEGs, longitudinal changes in PAF are described in Supplementary Table 10. Fig. S4 illustrates individual 1/f-removed spectra for all EEGs, categorized by age and group.

In exploratory analyses, we fit region-specific logistic generalized linear mixed-effects models to predict diagnostic group (AS vs. NT) from peak alpha frequency (PAF), controlling for age and including a subject-level random intercept. PAF emerged as a robust predictor in all regions: frontal (p < 0.001; AUC = 0.94), central (p < 0.001; AUC = 0.98), and occipital (p < 0.001; AUC = 0.95).

### 3.4 Validation of reduced peaks and PAF using prominence analyses

**Fig. 4:**
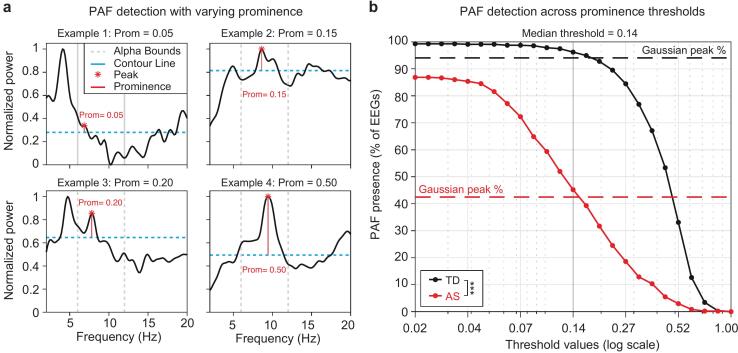
**Prominence detection confirms abnormal alpha oscillations in Angelman EEGs**. (a) Example spectra illustrating peaks at varying prominence levels. (b) Percentage of peaks detected in AS participants (red) vs. TD participants (black) across different threshold values. Dashed vertical lines represent the percentage of peaks detected for each group using Gaussian analysis, while the solid black vertical line indicates the median threshold (0.14). (For interpretation of the references to colour in this figure legend, the reader is referred to the web version of this article.)

Given the low rates of peak detection in AS spectra (Fig. 2**a-b**), we explored an alternative prominence-based peak detection method (Fig. 4**a**) to determine whether these low rates were influenced by Gaussian fitting process. Peak detection rates were consistently lower in AS participants compared to TD participants across all thresholds, including the most lenient (Fig. 4**b**). A linear mixed effects model demonstrated a significant effect of group (−3.8443, p < 0.001), indicating that participants in the AS group have significantly lower odds of peak detection compared to TD participants, and a significant effect of threshold (−12.389, p < 0.001), indicating that increasing threshold values are strongly associated with reduced odds of detecting a peak across all participants. The interaction effect between group and threshold (−0.0204, p = 0.973) was not statistically significant, indicating that the effect of threshold on peak detection does not differ significantly between AS and TD participants. This suggests that the absence of peaks in AS spectra was not due to the Gaussian fitting process being overly strict or failing to detect less distinct peaks in AS.

Peak detection rates at the median threshold (0.14) were 69.41 %, 76.45 %, and 72.22 % for the frontal, central, and occipital regions, respectively. These rates were consistent with those obtained using the Gaussian approach (68.24 %, 72.09 %, and 70.47 %). Cohen’s kappa statistics demonstrated substantial inter-rater agreement in peak detection rates between the two methods: frontal (κ = 0.70, 95 % CI [0.603, 0.767]), central (κ = 0.611, 95 % CI [0.516, 0.696]), and occipital (κ = 0.61, 95 % CI [0.522, 0.70]). For participants with a detected peak, PAF values were highly consistent between the two methods, with average differences of −0.38 Hz (frontal), −0.31 Hz (central), and −0.34 Hz (occipital). Intraclass correlation coefficients (ICCs) further confirmed the strong agreement between methods, with high consistency observed across all regions: ICC = 0.86 (frontal), ICC = 0.90 (central), and ICC = 0.78 (occipital), all p < 0.0001.

## 4 Discussion

Alpha oscillations undergo well-characterized developmental changes that closely align with neural circuit maturation, making them a promising focus for understanding atypical brain function. This study provides novel insights into alpha metrics, including PAF, to determine their value as markers for characterizing and mapping atypical neuronal dynamics in AS. Our results demonstrate that PAF is highly sensitive to loss of neuronal Ube3a expression, providing a potential marker to better understand both the underlying neuropathology and the unique developmental trajectories associated with AS.

Consistent with previous findings, we observed higher delta power and lower relative alpha power in children with AS (Fig. 1), indicating altered oscillatory dynamics. As shown in Fig. 1, the markedly greater within–group variance in delta power among AS participants likely reflects both their overall dominance of slow‐wave activity and individual differences in the persistence of dominant delta rhythms. Conversely, typically developing children have transitioned to an alpha-dominant profile, indicated by decreased delta and increased alpha power. However, the significant variability in alpha power emphasizes individual differences in the progression of these maturity markers rhythms. To further probe these dynamics, we quantified PAF and found that TD children consistently displayed pronounced alpha peaks and exhibited typical age-related increases in PAF. In contrast, developmental patterns were significantly disrupted in AS. Children with AS were significantly less likely to exhibit an alpha peak compared to age-matched typically developing controls (Fig. 2), and among those who did show an alpha peak, PAF was significantly lower (Fig. 3). Furthermore, while prominent in the TD group, the well-established age-related increase in PAF was absent in AS (Fig. 3, Fig. 4).

In addition, when PAF was observed in AS EEGs, the spectra appeared qualitatively different than in TD controls (Fig. 2**c-d**). The TD subjects consistently displayed clear, pronounced peaks in the alpha band, whereas PAF was decreased in both amplitude and frequency and appeared qualitatively “immature”, resembling spectra often seen in typically developing infants (Fig. 2**c**) (Marshall et al., 2002, Wilkinson et al., 2024). The examples shown in Fig. 2**c** are representative, and the less mature alpha profile consistent with earlier developmental stages was also observed across group averages of AS EEGs where PAF was determined to be present (Fig. 2**d**). Even when using a more sensitive prominence-based peak detection method, AS participants consistently exhibited lower peak rates compared to TD children, regardless of the threshold applied (Fig. 4). These consistent findings across detection methods suggest that the atypical alpha dynamics in AS reflect genuine neurophysiological differences rather than methodological artifacts.

### 4.1 Implications for alpha metrics in AS

These results highlight the potential of alpha oscillations, specifically the presence and frequency of an alpha peak, as critical markers for assessing brain function in AS. Specifically, alpha metrics are highly sensitive to atypical circuit function, making them potentially valuable for tracking responses to treatments aimed at improving brain function (Dickinson et al., 2018, Edgar et al., 2019). Given that alpha peaks were detectable in only 50 % of participants, tracking peak frequency alone would not capture neural dynamics in children without a clear peak; metrics quantifying alpha emergence or peak presence may therefore be more broadly applicable. Specifically, when combined with other measures, such as delta power, alpha metrics may provide a powerful framework for detecting changes in neural circuits targeted by disease-modifying treatments, offering insights that precede observable downstream behavioral improvements. For instance, alpha metrics may directly index maturational changes in the underlying large-scale circuits that support increasingly complex cognition and behavior (Klimesch et al., 2007, Marshall et al., 2002). Such early detection capabilities are critical for evaluating intervention efficacy and may help address the critical need for quantifiable biomarkers to support and enhance clinical endpoints. Furthermore, these EEG-based measures offer a unique opportunity to serve as translational biomarkers, providing a consistent framework for evaluating neural changes across both preclinical and clinical trials. This dual utility underscores their promise as tools for advancing therapeutic development and understanding the mechanisms underlying treatment effects in AS.

Alpha metrics may also complement delta metrics by providing distinct and complementary insights into neural function. Given that elevated delta power reflects generalized circuit disruption, therapeutic improvements in delta power (i.e., reductions) would likely reflect circuits becoming less disorganized and movement towards more typical circuit dynamics. This aligns with the observed age-related reductions in delta power, which may represent the natural progression of neural circuits maturing and transitioning toward more efficient states. In contrast, alpha metrics, such as PAF, may offer a finer-grained measure of how circuits are actively maturing and achieving greater functional specialization. Furthermore, because alpha metrics undergo rapid and measurable changes during early development as circuits mature (Dickinson et al., 2025, Wilkinson et al., 2024), they may be particularly well-suited for detecting fast, subtle changes in neural activity over the course of a clinical trial. PAF, in particular, is a valuable metric because it remains generally stable across behavioral conditions and is quantified in a way that is not influenced by increased delta (Dickinson et al., 2018, Grandy et al., 2013, Haegens et al., 2014, Petrosino et al., 2018). This sensitivity to developmental dynamics positions alpha metrics as powerful tools for tracking treatment effects and understanding neural mechanisms underlying variable trajectories in AS.

### 4.2 Limitations and next steps

While these findings underscore the significant potential of alpha metrics as sensitive markers of neural function and maturation, this study is not without limitations, and important next steps are needed to advance our understanding of alpha metrics in this context. One potential limitation is that EEGs from children with AS were collected using clinical systems, whereas EEGs from neurotypical controls were obtained from high-density research systems. Although this difference could introduce small variations in signal characteristics, the stark differences observed between the AS and NT groups are unlikely to be solely attributable to equipment variations. Additionally, both clinical and research systems are widely validated for capturing reliable neural signals in pediatric populations. Future studies with identical EEG systems across groups would further ensure methodological consistency; however, the pronounced findings here strongly suggest that the observed atypical alpha dynamics in AS reflect a true underlying neurophysiological difference rather than an artifact of system differences. Collecting AS data using high-density research-grade EEG systems and harmonized protocols would also enable analysis of additional neurophysiological metrics. For instance, due to differences in montage and electrode coverage, metrics such as functional connectivity were beyond the scope of the current dataset but could be fruitfully explored in future studies.

Another consideration is variation in EEG recording length across participants. While the clinical EEGs in the AS group were often longer, which may have enhanced signal reliability and sensitivity to detect alpha activity, shorter recordings in the NT group could limit the ability to detect more subtle features. Although we included recording length as a covariate in all models to account for this variability, future work with matched recording durations across groups would provide a more controlled assessment.

Another limitation of this study is that the age-related changes described are based on cross-sectional data, which provide a snapshot of developmental trends across different individuals but may not fully capture the dynamic trajectories of neural changes over time. Future work may leverage data from individuals with multiple data points to provide a more comprehensive report of longitudinal developmental PAF patterns in AS and how these changes map onto variations in cognitive function, and changes over time. In addition, while this study highlights alpha metrics as markers of neural maturation, further work is needed to investigate how these neural changes map onto cognitive development. Understanding the relationship between alpha oscillations and cognitive function is crucial for determining whether alpha metrics can serve as meaningful biomarkers of functional outcomes. This would provide critical context for interpreting their potential role in tracking developmental changes and assessing the efficacy of interventions in AS. Comparisons of PAF in deletion and non-deletion AS genotypes in this study provide important preliminary data: 74 % of non-deletion EEGs had a detectable alpha peak whereas 55 % of deletion EEGs had a detectable peak. This difference was not statistically significant (p = 0.12, Supplementary Table 6), but given the generally more severe clinical phenotype in deletion-positive AS (Gwaltney et al., 2024, Tjeertes et al., 2023, Yang et al., 2021), does point towards a potential avenue for future investigation.

Finally, as an initial characterization of alpha metrics, this study used the 6–12 Hz range to examine alpha dynamics, consistent with frequency ranges commonly applied in developmental populations. However, given the dysmaturation of alpha oscillations observed in our analyses, it is important to recognize the limitations of using fixed frequency boundaries during early spectral maturation. In typical development, spectral maturation involves a gradual reorganization of power across frequencies (Dickinson et al., 2025, Patino et al., 2017, Xiao et al., 2015). Therefore, adopting a developmental framework that captures these broader shifts in power may offer a more flexible and accurate approach for quantifying spectral dynamics in AS, which deviates significantly from age-expected patterns of change. Future work should explore frequency ranges that are not strictly predefined, enabling a more comprehensive and sensitive assessment of neural maturation and atypical development in AS. A broad-spectrum marker would continuously capture evolving oscillatory dynamics, providing a robust tool for monitoring developmental and treatment effects across varied stages of maturation.

## CRediT authorship contribution statement

**Abigail H. Dickinson:** Writing – review & editing, Writing – original draft, Visualization, Validation, Supervision, Software, Methodology, Funding acquisition, Formal analysis, Data curation, Conceptualization. **M.Sapphire Bowen-Kauth:** Writing – review & editing, Writing – original draft, Visualization, Resources, Investigation, Formal analysis, Data curation. **Jeremy J. Shide:** Writing – review & editing, Writing – original draft, Visualization, Resources, Investigation, Formal analysis, Data curation. **Anna E. Youngkin:** Writing – review & editing, Resources, Investigation, Formal analysis, Data curation. **Nishitha S. Hosamane:** Writing – review & editing, Resources, Investigation, Formal analysis, Data curation. **Courtney A. McNair:** Writing – review & editing, Resources, Investigation, Formal analysis, Data curation. **Declan P. Ryan:** Writing – review & editing, Resources, Investigation, Data curation. **Catherine J. Chu:** Writing – review & editing, Resources, Investigation, Data curation. **Michael S. Sidorov:** Writing – review & editing, Writing – original draft, Visualization, Supervision, Resources, Methodology, Investigation, Funding acquisition, Formal analysis, Data curation, Conceptualization.

## Funding

This work was supported by the 10.13039/100009726Foundation for Angelman Syndrome Therapeutics [FT2002-003].

## Declaration of competing interest

The authors declare that they have no known competing financial interests or personal relationships that could have appeared to influence the work reported in this paper.

## Data Availability

The authors do not have permission to share data.
