# Supplementary material for: Atypical alpha oscillatory EEG dynamics in children with Angelman syndrome
Source: Neuroimage Clin. 2025 Aug 13;48:103865. doi: 10.1016/j.nicl.2025.103865 (PMC12409793; doi:10.1016/j.nicl.2025.103865)
Supplement: Supplementary Data 1 [file mmc1.docx]

| Number EEGs Contributed | Number of participants |
| --- | --- |
| 1 EEG | 60 |
| 2 EEGs | 16 |
| 3 EEGs | 12 |
| 4 EEGs | 5 |
| 5 EEGs | 1 |
| 6 EEGs | 1 |

**Supplementary Table 1.** Number of AS EEGs contributed per participant.

|  | **AS EEGs** | |
| --- | --- | --- |
|  | *Site 1 (UNC)* | *Site 2 (ASNHS)* |
| N EEGs | 10 | 149 |
| Age (y) | 5.1 ± 1.2 | 6.2 ± 0.3 |
| Sex | M: 5 (50%)  F: 5 (50%) | M: 97 (65%)  F: 52 (35%) |
| EEG system | Natus or Grass | Bio-Logic or Xltek |
| N Channels | 6 (F3, F4, C3, C4, O1, O2) | 10-20 system |
| Sampling rate (Hz) | 200 or 256 | 200, 256, or 512 |
| Recording length (min) | 76.8 ± 18.5 | 21.2 ± 1.5 |
| Reference | Vertex | Varied |

**Supplementary Table 2.** Site-by-site properties of AS EEGs.

|  | **TD EEGs** | | |
| --- | --- | --- | --- |
|  | *Site 1 (UNC)* | *Site 2 (MGH)* | *Site 3 (UCLA)* |
| N EEGs | 10 | 98 | 77 |
| Age (y) | 5.8 ± 1.3 | 7.5 ± 0.3 | 3.7 ± 0.3 |
| Sex | M: 5 (50%)  F: 5 (50%) | M: 52 (53%)  F: 46 (47%) | M: 42 (55%)  F: 35 (45%) |
| EEG system | Natus or Grass | Natus | EGI |
| N Channels | 6 (F3, F4, C3, C4, O1, O2) | 10-20 system | 129-channel HydroCel Geodesic Sensor net |
| Sampling rate (Hz) | 200 or 256 | 200, 250, 256, 500, or 512 | 250 |
| Recording length | 67.3 ± 12.3 | 10.6 ± 1.3 | 2.5 ± 0.2 |
| Reference | Vertex | C2 spinous process | Vertex |

**Supplementary Table 3.** Site-by-site properties of TD EEGs.

| Predictor | Estimate | SE | t | df | p | 95% CI (Lower) | 95% CI (Upper) | OddsRatio |
| --- | --- | --- | --- | --- | --- | --- | --- | --- |
| (Intercept) | -1.804 | 0.423 | -4.267 | 1023 | 2.164E-05 | -2.633 | -0.974 | 0.165 |
| NT group (vs. AS) | 3.589 | 0.653 | 5.493 | 1023 | 4.983E-08 | 2.307 | 4.871 | 36.183 |
| Age | 0.0179 | 0.00403 | 4.448 | 1023 | 9.635E-06 | 0.01 | 0.0258 | 1.018 |
| Recording Length | -5.953E-05 | 8.866E-05 | -0.671 | 1023 | 0.502 | -0.000234 | 0.000114 | 1 |
| Region_Central | 0.372 | 0.274 | 1.36 | 1023 | 0.174 | -0.165 | 0.909 | 1.451 |
| Region_Occipital | 0.187 | 0.274 | 0.684 | 1023 | 0.494 | -0.35 | 0.725 | 1.206 |
| Group X Age | -0.000927 | 0.00811 | -0.114 | 1023 | 0.909 | -0.0168 | 0.015 | 0.999 |
| Group x Region_Central | 0.335 | 0.566 | 0.592 | 1023 | 0.554 | -0.776 | 1.446 | 1.398 |
| Group x Region_Occipital | 0.248 | 0.544 | 0.455 | 1023 | 0.649 | -0.82 | 1.315 | 1.281 |

**Supplementary Table 4.** Reduced PAF presence in AS.

|  | Estimate | SE | t | df | p | 95% CI (Lower) | 95% CI (Upper) |
| --- | --- | --- | --- | --- | --- | --- | --- |
| (Intercept) | -0.934 | 0.531 | -1.757 | 145 | 0.081 | -1.984 | 0.117 |
| Age | 0.232 | 0.068 | 3.422 | 145 | 0.001 | 0.098 | 0.366 |
| RecordingLength_sec_ | -0.0002 | 0.0002 | -0.889 | 145 | 0.376 | -0.001 | 0.0002 |
| Neuroactive_Meds_Yes | 0.346 | 0.492 | 0.704 | 145 | 0.483 | -0.626 | 1.318 |

**Supplementary Table 5.** Evaluating PAF presence as a function of neuroactive medication.

|  | Estimate | SE | t | df | p | 95% CI (Lower) | 95% CI (Upper) |
| --- | --- | --- | --- | --- | --- | --- | --- |
| (Intercept) | -0.887 | 0.446 | -1.989 | 146 | 0.0486 | -1.769 | -0.00561 |
| Age | 0.224 | 0.066 | 3.390 | 146 | 0.000898 | 0.0933 | 0.354 |
| RecordingLength_sec_ | -0.000108 | 0.000157 | -0.691 | 146 | 0.490 | -0.000418 | 0.000202 |
| Genotype (nonDeletion vs deletion) | 0.765 | 0.492 | 1.555 | 146 | 0.122 | -0.208 | 1.738 |

**Supplementary Table 6.** Evaluating PAF presence as a function of AS genotype.

|  | Factor | Estimate | SE | t | df | p | 95% CI (Lower) | 95% CI (Upper) |
| --- | --- | --- | --- | --- | --- | --- | --- | --- |
| Frontal | Intercept | 6.91 | 0.308 | 22.425 | 227 | 1.71E-59 | 6.304 | 7.519 |
|  | Group | 0.444 | 0.334 | 1.33 | 227 | 0.185 | -0.214 | 1.102 |
|  | Age | 0.0148 | 0.0361 | 0.41 | 227 | 0.682 | -0.0564 | 0.0859 |
|  | Recording Length | -1.73E-05 | 5.30E-05 | -0.327 | 227 | 0.744 | -0.000122 | 8.71E-05 |
|  | Group *Age | 0.152 | 0.0416 | 3.643 | 227 | 0.000334 | 0.0696 | 0.234 |
| Central | Intercept | 6.788 | 0.235 | 28.922 | 243 | 1.20E-80 | 6.326 | 7.251 |
|  | Group | 0.746 | 0.259 | 2.879 | 243 | 0.00435 | 0.236 | 1.257 |
|  | Age | 0.00822 | 0.0282 | 0.292 | 243 | 0.771 | -0.0473 | 0.0637 |
|  | Recording Length | 2.87E-05 | 4.18E-05 | 0.687 | 243 | 0.493 | -5.36E-05 | 0.000111 |
|  | Group *Age | 0.176 | 0.033 | 5.343 | 243 | 2.10E-07 | 0.111 | 0.241 |
| Occipital | Intercept | 6.486 | 0.229 | 28.375 | 236 | 5.104E-78 | 6.0355 | 6.936 |
|  | Group | 0.738 | 0.256 | 2.88 | 236 | 0.00435 | 0.233 | 1.244 |
|  | Age | 0.0736 | 0.0278 | 2.645 | 236 | 0.00872 | 0.0188 | 0.128 |
|  | Recording Length | 1.292E-05 | 4.692E-05 | 0.275 | 236 | 0.783 | -7.951E-05 | 0.000105 |
|  | Group *Age | 0.152 | 0.0331 | 4.603 | 236 | 6.804E-06 | 0.0872 | 0.218 |

**Supplementary Table 7.** Atypical developmental PAF trajectory in AS. For this analysis, categorical predictors were effect-coded with AS group as the reference for Group, and Frontal region as the reference for region.

|  | Estimate | SE | t | df | p | 95% CI (Lower) | 95% CI (Upper) |
| --- | --- | --- | --- | --- | --- | --- | --- |
| (Intercept) | 6.546 | 0.271 | 24.12 | 86 | <.001 | 6.007 | 7.086 |
| Age | 0.035 | 0.027 | 1.29 | 86 | 0.2 | -0.019 | 0.09 |
| RecordingLength_sec_ | 0.0001 | 0.0001 | 0.991 | 86 | 0.325 | -0.0001 | 0.0003 |
| Neuroactive_Meds_Yes | 0.023 | 0.231 | 0.101 | 86 | 0.92 | -0.436 | 0.482 |

**Supplementary Table 8.** Evaluating peak alpha frequency as a function of medication.

|  | Estimate | SE | t | df | p | 95% CI (Lower) | 95% CI (Upper) |
| --- | --- | --- | --- | --- | --- | --- | --- |
| (Intercept) | 6.552 | 0.235 | 27.931 | 87 | 3.281E-45 | 6.086 | 7.019 |
| Age | 0.0395 | 0.027 | 1.462 | 87 | 0.147 | -0.0142 | 0.0931 |
| RecordingLength_sec_ | 1.346E-05 | 7.354E-05 | 0.183 | 87 | 0.855 | -0.000133 | 0.00016 |
| Geno_NonDeletion | 0.181 | 0.204 | 0.886 | 87 | 0.378 | -0.225 | 0.587 |

**Supplementary Table 9.** Evaluating peak alpha frequency as a function of AS genotype.

**Reported Medications**

Medication data were available for the subsample from Site 2 (ASNHS), comprising 149 of 159 Angelman Syndrome (AS) EEG recordings from 88 unique participants. Of the unique participants, 12/88 reported no medications, and 22 reported no neuroactive meds (but potentially using supplements/GI medications, etc). Across the dataset, participants reported a total of 79 unique medications and supplements, grouped below by their functional categories:

| **Medication Class** | **Medications** |
| --- | --- |
| Anti-Epileptic Drugs (AED) | Valproic acid, Depakene, Lamotrigine, Keppra, Topiramate, Phenobarbital, Ethosuximide, Carbamazepine, Phenytoin, Vigabatrin, Zarontin, Zonegran, Clobazam, Diastat |
| Benzodiazepines/Sedatives | Klonopin, Diazepam, Lorazepam, Ativan, Tranxene, Trazodone, Gabapentin, Neurontin, Baclofen, Hydroxyzine, Theanine |
| Sleep-Aids/OTC Sedatives | Melatonin, Diphenhydramine, Benadryl |
| Stimulants (ADHD medications) | Ritalin |
| Antipsychotics/Mood Stabilizers | Risperdal |
| Dopaminergic Agents | Levodopa/Carbidopa |
| Respiratory/Allergy Medications | Albuterol, Pulmicort, Flovent, Qvar, Xopenex, Singulair, Claritin, Zyrtec, Nasonex, Levocetirizine, Cetirizine |
| Gastrointestinal (GI)/Laxative-Reflux Medications | Prilosec, Omeprazole, Prevacid, Zegerid, Pepcid, Zantac, Ranitidine, Miralax, Dulcolax, Ex-lax, Benefiber, Laxative/Stool softener, Propulsid, Reglan, Nexium |
| Supplements/Nutritive Agents | Actifolate, Betaine, Carnitine, Creatine, Coenzyme Q10, Folic Acid, Metafolin, Omega-3, Vitamin B12, Argentum Bryophyllum, Aurum Prunus, Calcium, Calms-Forte, Curcumin, Fatty acids (including Omega-3), Fish Oil, Fluoride, Iron, Methyl B12, Multivitamin, Polyvisol, Probiotics, Vitamins B, C, D, Vitamin B6 Complex, Vitamin B6, Vitamin B6-B12-Folic Acid, Vitamin D |
| Other/Non-Neuroactive Medications | Advil, Antibiotics, Atopiclair, Cyproheptadine, Depo-Provera, Desonide, Glycopyrrolate, H-Cortisone-Iodoquinol, Hydrocortisone, Lantus, Levocarnitine, Motrin, Zofran |

**Analysis of Medication Effects**

We examined the potential effect of neuroactive medications, defined as compounds primarily affecting central nervous system (CNS) function, including AEDs, benzodiazepines/sedatives, sleep aids, stimulants, antipsychotics, and dopaminergic agents. Specifically, we examined whether neuroactive medications were associated with 1) the presence or absence of an alpha peak, or 2) differences in the frequency of identified alpha peaks.

Medication data were available for 149 recordings; 33 recordings were completed without any neuroactive medication, while 116 recordings included at least one neuroactive medication. To analyze the effect of medication status on peak presence, we used a general linear mixed-effects logit model controlling for age and recording length, with a random subject intercept (Supplementary Table 5).

Among participants with an identified peak, we employed a generalized mixed model (GMM) to examine the relationship between neuroactive medication status and average alpha peak frequency, again controlling for age, recording length, and including a random subject intercept (Supplementary Table 8).

Our analyses indicated no significant differences in peak presence or peak frequency associated with neuroactive medication use. We conducted additional analyses comparing participants on anti-epileptic drugs versus those without, and those on benzodiazepines versus those without; no significant differences emerged from these analyses.

**Analysis of Genetic Subtypes**

Genetic subtype information was available for 89 out of 95 AS participants, corresponding to 150 of the 159 EEG recordings. A range of genetic subtypes was observed, as detailed in Figure S1B. To investigate potential differences in alpha peak characteristics by genetic subtype, we categorized participants into deletion and non-deletion groups.

We conducted analyses analogous to the medication analysis to examine whether genotype subtype influenced peak presence. Among the 150 EEG recordings with genotype information, 107 were from deletion cases and 43 from non-deletion cases. Although differences did not reach statistical significance (p = 0.12), there was an indication that non-deletion participants were more likely to exhibit an alpha peak: 74% of non-deletion cases had a detectable peak in any region compared to 55% of deletion cases (Supplementary Table 6). However, a GLMM examining potential peak frequency differences between deletion and non-deletion cases did not indicate differences by genetic subtype (p=0.37; See Supplementary Table 9).

**Data Processing Functions and Parameters**

% Filtering

function EEG_filt = threefilters_NH(unfiltData, SamplingRate)

data_filtered = unfiltData;

% Filtering parameters

params.highpass = 1; % Digital highpass filter 1 Hz

params.lowpass = 99.5; % Digital lowpass filter 100 Hz

params.notch = [59 61]; % Notch filter parameters, 60 Hz notch.

params.butter = 2; % Second order filters.

% ----- Highpass -----

Wn = params.highpass/(SamplingRate/2);

[b,a] = butter(params.butter,Wn,'high');

data_filtered = filter(b,a,data_filtered);

% ----- Lowpass -----

Wn = params.lowpass/(SamplingRate/2);

[b,a] = butter(params.butter,Wn,'low');

data_filtered = filter(b,a,data_filtered);

% ----- Notch -----

Wn = params.notch/(SamplingRate/2);

[b,a] = butter(params.butter,Wn,'stop');

data_filtered = filter(b,a,data_filtered);

EEG_filt = data_filtered;

Basics of spectral analysis:

[s,w,t, pxx] = spectrogram(channel.values, params.spec.window, params.spec.noverlap, params.spec.nfft, SaRa); % Generate spectrogram on active channel with params defined. pxx is the matrix holding the spectrogram data.

xx.freq = pxx(params.spec.freq_min_bins:params.spec.freq_max_bins,:); % Extracts the relevant rows of the spectrogram

xx.total = pxx(params.spec.total_min_bins:params.spec.total_max_bins,:);
