# Supplementary figures and images for: Atypical alpha oscillatory EEG dynamics in children with Angelman syndrome
Source: Neuroimage Clin. 2025 Aug 13;48:103865. doi: 10.1016/j.nicl.2025.103865 (PMC12409793; doi:10.1016/j.nicl.2025.103865)

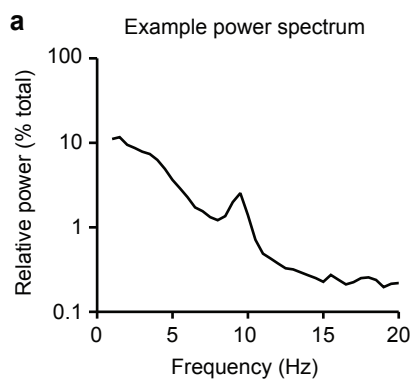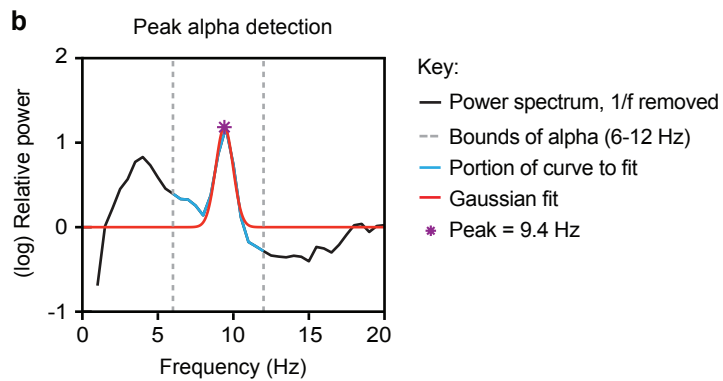

Figure S2

Supplement: Supplementary Data 2 [file mmc2.pdf]

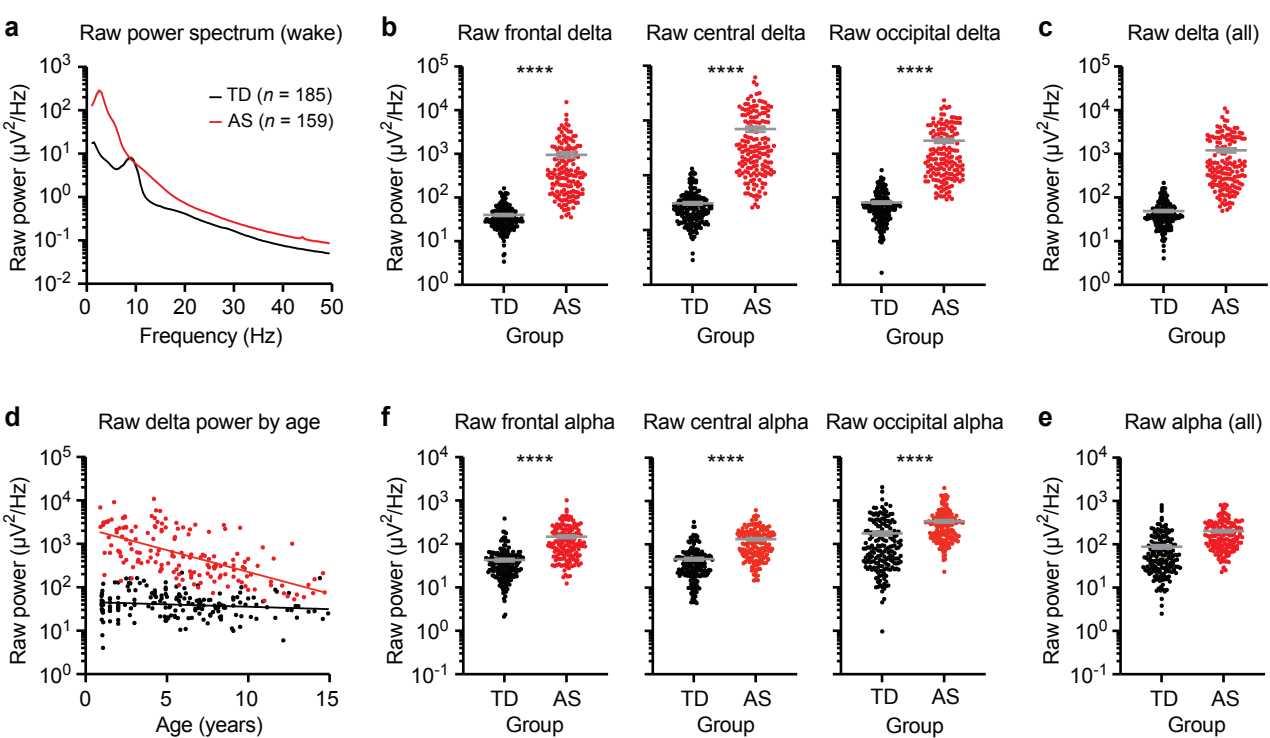

Figure S3

Supplement: Supplementary Data 3 [file mmc3.pdf]

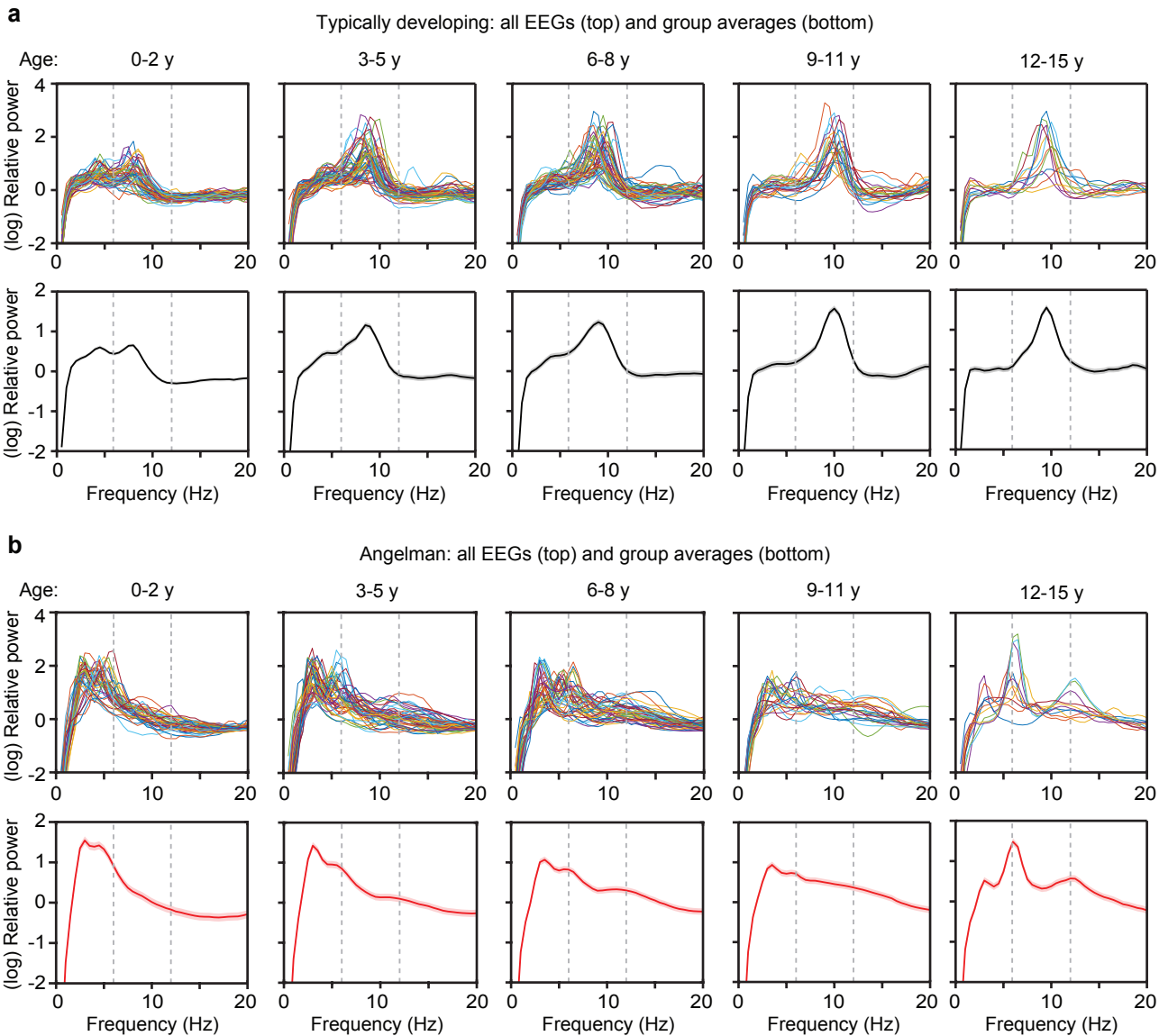

Figure S4

Supplement: Supplementary Data 4 [file mmc4.pdf]
